# Electronic Structure and Redox of the Antidepressants Venlafaxine and Desvenlafaxine

**DOI:** 10.1021/acsomega.5c08632

**Published:** 2025-11-25

**Authors:** Jhon Kennedy Alves Pereira, Eufrásia de Sousa Pereira, Bárbara Júlia Gonçalves Dutra, Isaac Yves Lopes de Macêdo, Arthur Saldanha Guimarães, Bruno Junior Neves, Eric de Souza Gil, Freddy Fernandes Guimarães

**Affiliations:** † Laboratory of Pharmaceutical and Environmental Analysis, Faculdade de Farmácia, 67824 Universidade Federal de Goiás, Goiânia 74605-170, Goiás, Brazil; ‡ Laboratory of Cheminformatics, Faculdade de Farmácia, Universidade Federal de Goiás, Goiânia 74605-170, Goiás, Brazil; § Institute of Chemistry, Universidade Federal de Goiás, Goiânia 74605-170, Goiás, Brazil

## Abstract

Venlafaxine and its primary metabolite desvenlafaxine are antidepressants that block presynaptic reuptake of serotonin and norepinephrine in the brain. Electroanalytical and computational analyses were performed to evaluate the electrochemical characterization of these drugs through measurements using a carbon paste electrode alongside quantum calculations (DFT and TD–DFT) to support the electrochemical data and propose potential oxidation pathways. The results showed that both venlafaxine and desvenlafaxine exhibit different pH-dependent electrochemical behaviors, with desvenlafaxine showing higher anodic peak intensities at neutral pH, while venlafaxine peaks at alkaline pH. Computational insights from DFT calculations provided a deeper understanding of the molecular charge distribution, orbital profiles, and energetics of both drugs in neutral and protonated states. The Gibbs free energy variations in different medium environments revealed the critical role of the medium in modulating the thermodynamic stability. These findings presented here improve our understanding of the electrochemical and electronic properties of these antidepressants and may pave the way for the development of more effective therapeutic agents.

## Graphical abstract

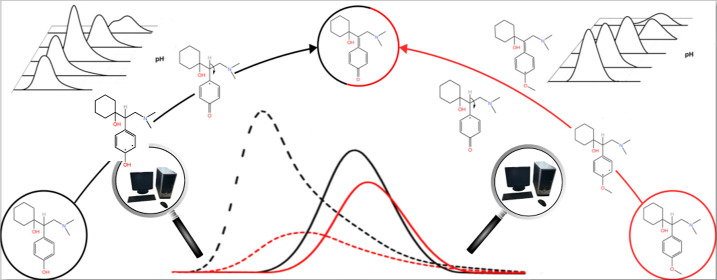

## 1 Introduction

Depression has been pointed out as a disease of modernity,^1^ and it significantly affects individuals and society as a whole. The World Health Organization estimates that worldwide 5% adults suffer from depression, making this disease a public health problem.^2^ Adverse social factors, such as structural inequalities, social isolation, poverty, and lack of access to healthcare, have an impact on mental health.^3,4^ There is evidence that stressful conditions can lead to oxidative stress, a condition in which the body’s antioxidant defenses are overwhelmed by high levels of reactive oxygen species (ROS). This means that ROS levels can play a role in the pathology of depression.^5^ For instance, stress related to elevated ROS levels can compromise neurotransmitter production by oxidizing tetrahydrobiopterin, an essential coenzyme involved in neurotransmitter biosynthesis. This indicates that compounds with antioxidant profiles can be used to inhibit depressive stress.^6,7^

Among the drugs used to treat depression, venlafaxine (VEN) and its main metabolite desvenlafaxine (DES) act by inhibiting serotonin and noradrenaline reuptake inhibitors (SNRI).^8–10^ Despite similar neuropharmacological efficiency,^8^ their ability to protect cellular damage may be dictated by the antioxidant action on lipid peroxidation.^11^ In this case, experimental electroanalysis can provide information on the kinetics of the redox reaction, reversibility, and the electron–proton transfer correlation structure.^11^ Computational chemistry can be applied to predict various biological and chemical parameters through molecular modeling. One such parameter, the electronegativity value, is directly related to redox processes. Redox properties are also related to molecular orbital parameters and molecular geometry. Electronic structure quantum chemistry calculations, such as density functional theory (DFT), are excellent tools^12^ that provide data such as molecular geometry, atomic charges, orbital energies, and their spatial distributions, among other applications. Due to the direct correlation between antioxidant activity and the therapeutic effect, the evaluation and correlation of these parameters can provide valuable information on the chemical components and processes responsible for the biological effect.

In this work, a detailed study of venlafaxine (VEN) and desvenlafaxine (DES) is performed throughout computational calculations and electroanalytical techniques. The DFT calculations provided detailed information on the electronic distribution of molecular orbitals and their energetic profile, leading to maps of Molecular Electronic Potentials (MEP) depicting the molecular charge distribution. The theoretical results for the VEN and DES consider both the neutral and protonated forms of the molecules in addition to the proposed product of the oxidation. The time dependent density functional theory (TD–DFT) was employed to give insights about single excitations for the 3 first excited electronic states. The redox behavior of VEN and DES was measured by voltammetric analysis to evaluate the redox profile and analyzed in light of the DFT and TD–DFT results. The neutral, protonated, and oxidized forms of VEN and DES are compared to reveal conserved features and contrasting properties.

## 2 Theoretical and Experimental Methods

### 2.1 Materials and Reagents

All chemicals and solvents used were reagent grade, and they were used without further purification. Double-distilled Milli-Q water (conductivity ≤0.1 μS cm^–1^) (Millipore S. A., Molsheim, France) was used as a solvent for all solutions. Venlafaxine and desvenlafaxine were purchased from Sigma (St. Louis, MO), and the corresponding stock solutions were prepared immediately prior to experiments.

### 2.2 Electrochemical Measurements

Voltammetric experiments were carried out with a potentiostat/galvanostat mAutolab III integrated into GPES 4.9 software (EcoChemie, The Netherlands). Electrochemical measurements were performed in a 5.0 mL one-compartment cell, with a three-electrode system consisting of a carbon past electrode (CPE), a Pt wire, and Ag/AgCl/KCl (sat.) (Lab solutions, Brazil) representing the working, neutral, and reference electrodes, respectively. All experiments were carried out in triplicate at room temperature (25 ± 1 °C). 0.1 M acetate buffer solutions (ABS) and/or phosphate buffer solutions (PBS) were used as an electrolyte by adding 0.1 mM solutions of HCl or NaOH.

The following experimental conditions were applied: A pulse amplitude of 50 mV, pulse width of 0.2 s and scan rate 25 mV s^–1^ in the differential pulse voltammetry (DPV). In square wave voltammetry (SWV) was used a pulse amplitude of 50 mV, frequency 25 Hz, and a potential increase of 2 mV (corresponding to a scan rate of 50 mV s^–1^). In cyclic voltammetry (CV), scan rates of 25, 50, 100, 250, and 500 mV s^–1^ were applied that ranged from 0 to 1.1 V. The differential pulse voltammograms were subtracted from the background and corrected for the baseline, and then all data were analyzed and treated with Origin 8 software.

### 2.3 Electronic Structure Calculations

The electronic structure calculations were divided into three stages. In the first stage, the electronic density distribution and the energy difference between the highest occupied molecular orbital (HOMO) and the lowest unoccupied molecular orbital (LUMO) were evaluated. During this phase, electronic structure calculations and molecular geometry optimizations with vibrational analysis were performed at the level of density functional theory (DFT).^13^ In the second stage, the wavelength of electronic transitions and oscillator strengths were determined by time-dependent density functional theory (TD–DFT).^14^ In both calculations, the def2-TZVP base set^15^ and the M06-2x^16^ exchange-correlation functional were used. These calculations were performed with Gaussian 16.^17^

In the third stage, the spontaneity of the demethylation process was investigated. The ORCA computational package^18^ was used in these calculations. Geometry optimizations and vibrational frequency calculations were performed using DFT with the wB97X-D3 ^19,20^ exchange–correlation functional and the def2-TZVP base sets together with the auxiliary def2-TZVP/J^15,21^ for all atoms. The solvent effect was included using the Truhlar and Cramer SMD model,^22^ as implemented in the ORCA program, where the electrostatic contribution to the energy is obtained using the Conductor-like Screening Model (COSMO) by Klamt.^23^

The total Gibbs free change in the gas phase, Δ*G*_gas_, was evaluated using

$$\Delta G_{gas}=\sum_{i}{\left(E_{elec-nucl}+G_{term}\right)}_{i,P}-\sum_{i}{\left(E_{elec-nucl}+G_{term}\right)}_{i,R}$$

where *E*_elec–nucl_ is the nuclear electronic energy of the species and *G*_term_ is the thermal contribution to Gibbs free energy, obtained within the harmonic oscillator and rigid rotor approach. The solvent effect energy change in solution was computed by

$$\Delta G_{sol}=\sum_{i}{\left(\Delta G_{P}^{ENP}+\Delta G_{P}^{CDS}\right)}_{i}-\sum_{i}{\left(\Delta G_{R}^{ENP}+\Delta G_{R}^{CDS}\right)}_{i}$$

where Δ*G* ^ENP^ represents the electrostatic contribution to the change in free energy, while Δ*G* ^CDS^ is the cavity term. The summations are performed over all *i*-th reactant species (R) and products (P), at the standard temperature of 298.15 K.

The oxidation and formation of the ketone group in the products can occur during an interaction of desvenlafaxine or venlafaxine with water (H_2_O) or hydroxyl ion (OH^–^). The overall process can be expressed as follows;

For desvenlafaxine

$$DES+nX\to ketone+nH_{2}O/H_{3}O^{+}$$

For venlafaxine

$$VEN+nX\to ketone+nH_{2}O/H_{3}O^{+}+{CH}_{3}OH$$

The *X* in the above chemical equations denotes (H_2_O) or the hydroxyl ion (OH^–^). Depending on the nature of the reagent *X*, either H_2_O or (H_3_O^+^) is generated. Specifically, in the case of venlafaxine, methanol (CH_3_OH) is additionally produced. The exact nature of *X* (whether it is water or hydroxyl ion) determines the specific products formed by the reaction.

For a detailed characterization of the thermodynamic stability and reactive profile of the studied species, we evaluated global electronic descriptors, namely: chemical potential (μ), global hardness (η), and electrophilicity index (ω). These parameters were derived from the energies of the frontier molecular orbitals (HOMO and LUMO), in accordance with Koopmans’ theorem for closed-shell systems, providing a direct connection between electronic properties and reactive behavior. The chemical potential μ reflects the tendency of a system to lose electron density, serving as an indicator of its relative electron affinity, while the global hardness η quantifies the intrinsic resistance of the system to electron redistribution, providing a measure of its robustness against charge transfer. These parameters are obtained using the expressions^24^

$$\mu =\frac{\left(\epsilon_{HOMO}+\epsilon_{LUMO}\right)}{2}$$

$$\eta =\frac{\left(\epsilon_{LUMO}-\epsilon_{HOMO}\right)}{2}$$

Complementarily, the electrophilicity index is defined as

$$\omega =\frac{\mu^{2}}{2\eta }$$

quantifies the ability of a species to accept electrons, representing the energy gain from electronic stabilization when the species reaches charge saturation from a reservoir of zero chemical potential.

## 3 Results and Discussion

The molecular geometries, the MEP boundaries, and the spatial distribution of the frontier molecular orbitals of desvenlafaxine and venlafaxine in neutral and protonated states are shown in Figure 1. The first row (1st) presents the superposition of molecular geometries; in A and C are the superpositions between the neutral and protonated species in the gas phase, and in B and D are the superpositions of the experimental crystallographic structures^25,26^ with theoretical results at the DFT/def2-TZVP/M062x level of theory. The values of the Root Mean Square Deviation (RMSD) of the atomic positions between the experimental and theoretical results are 0.4341 Å for the venlafaxine and 0.1860 Å for the desvenlafaxine. Such values represent a good agreement between the solid-state (crystallographic) and gas-phase (theoretical) molecular geometries. Furthermore, the corresponding absolute and relative errors for each atomic position are reported in Table 1. The neutral and protonated venlafaxine have an RMSD of 0.4377 Å, and the neutral and protonated desvenlafaxine have an RMSD of 0.1037 Å. These small values of RMSD indicate that the molecular protonation has little influence on the molecular geometry of these molecules. All of the RMSD values were computed without taking into account the hydrogen atoms. The column E is the product of oxidation alone.

**Figure 1:**
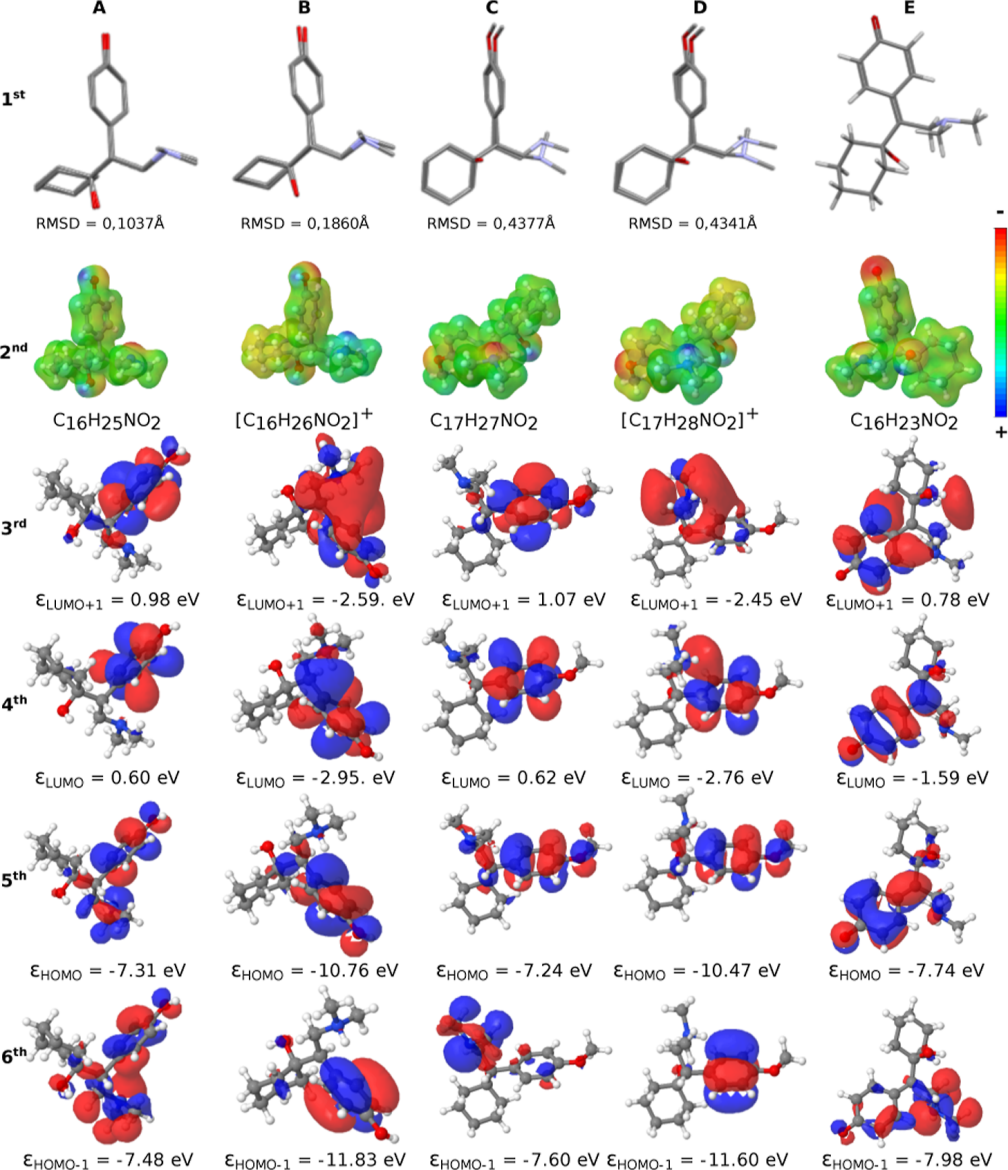
Superposition of the geometrical molecular structures (1st line), MEP boundaries (2nd line), spatial distribution of molecular orbitals (3rd-6th lines) for neutral and protonated states of desvenlafaxine (1st and 3rd columns) and venlafaxine (2nd and 4th columns), respectively, and their oxidation product (5th column). A and C show the overlay of the neutral and protonated forms of desvenlafaxine and venlafaxine. B and D are the superposition between the experimental crystallographic and DFT optimized geometrical structures. The hydrogen atoms were omitted in the molecular structure superpositions and in the Root-mean-square deviation (RMSD) calculations.

**Table 1:**
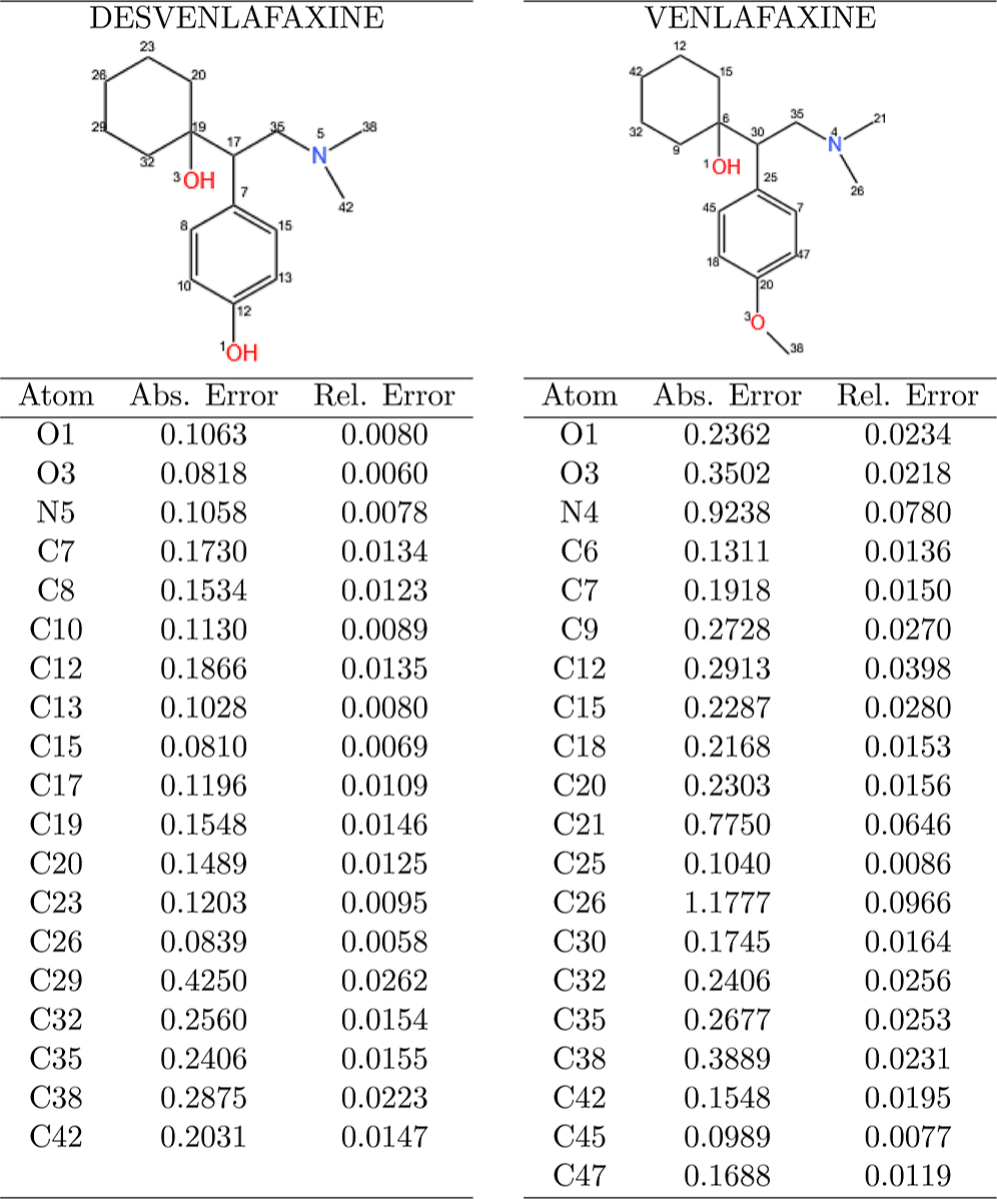
Absolute and Relative Errors of Atomic Positions for Desvenlafaxine (DES) and Venlafaxine (VEN)^a^

a The Hydrogen atoms were suppressed in the both calculations.

The molecular electrostatic potential (MEP) surfaces are shown in the second row (2nd) of Figure 1. The MEP surfaces are represented by colors that range from blue, representing regions deficient in electrons (+), to red, representing areas rich in electrons with a partial negative charge (−), as indicated by the added color bars. The MEPs demonstrate partial negative charges on oxygens for neutral and protonated molecules. Neutral species also have partial negative charges on the nitrogen atoms, while protonated species have a partial positive charge on the extra hydrogen bonded to the N atom. Small, partial positive charges are observed on the hydrogens of the hydroxyl group. The third, fourth, fifth, and sixth rows of Figure 1 show the frontier orbitals LUMO+1, LUMO, HOMO, and HOMO–1, respectively. For all species, the frontier orbitals LUMO+1, LUMO, HOMO, and HOMO–1 are located on the aromatic ring. The occupied molecular orbitals that are not located in the aromatic ring correspond to HOMO–2 for the neutral species, HOMO–3 for the protonated species, and HOMO–4 for the oxidation product (see Figures S5–S7 in Supporting Information). These orbitals are localized on the cyclohexane moiety and adopt a boat conformation in all studied cases.

Electrochemical characterization was performed through voltammetric analysis for desvenlafaxine and venlafaxine. The voltammograms were obtained at a pH ranging from 3 to 9. The redox profile of the drugs DES and VEN and their dependence on pH can be seen and compared in Figure 2. With respect to the dependence of pH, both DES and VEN showed an increase in overpotential requirements for the oxidation phenomenon as the pH became more acidic. Linearity was observed for both compounds between the anodic maximum potential and the pH, with a correlation coefficient of 0.98 for both molecules. The slope of the lines, close to 60 mV, top panels of Figure 2A,B, indicates a one-to-one electron–proton transference correlation, suggesting a specific oxidation mechanism for both molecules.

**Figure 2:**
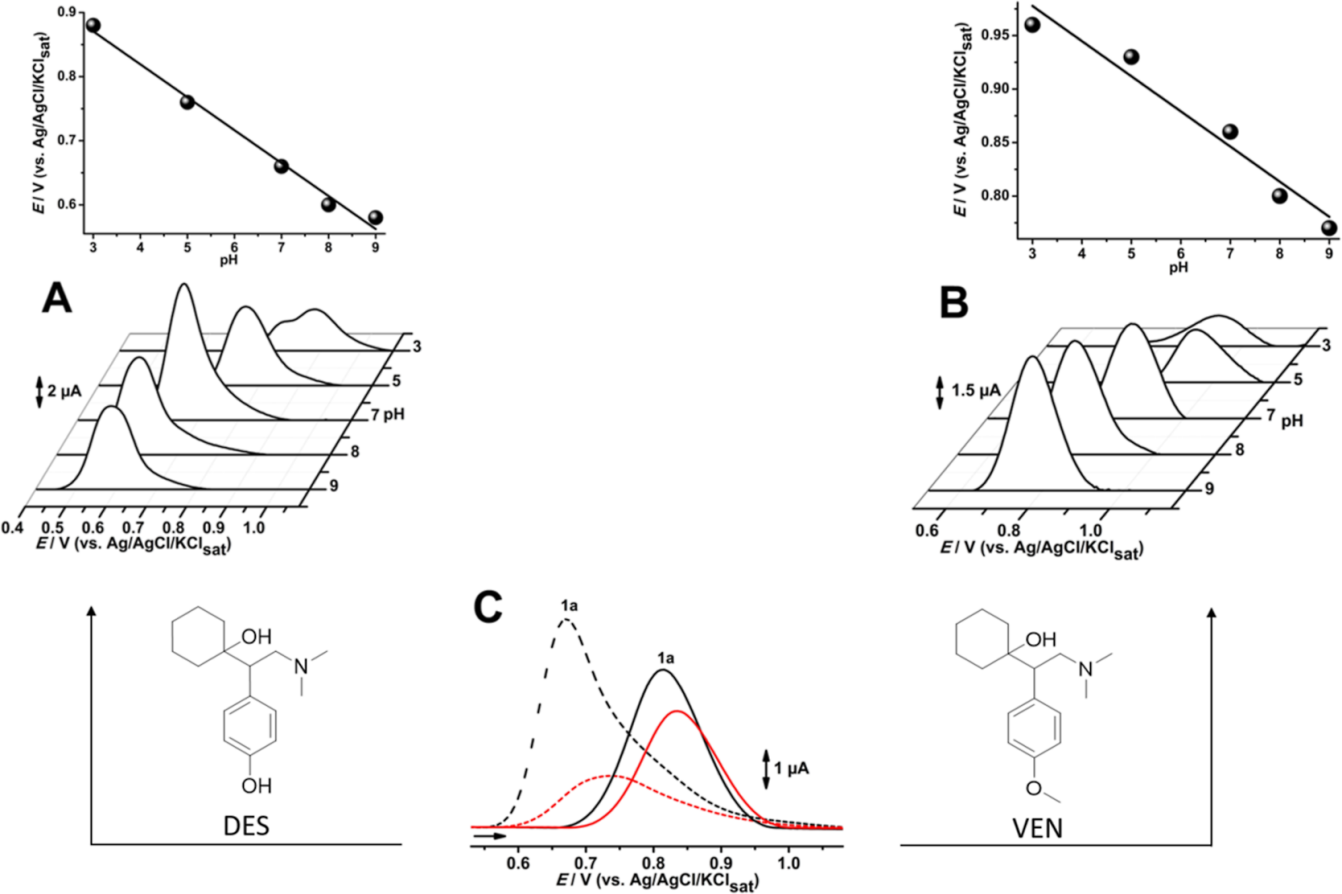
DP voltammograms (lower panels) and oxidation potential versus solution pH ranging from 3 to 9 (top panels) in 0.1 M ABS and/or PBS for DES (A) and VEN (B). C middle panel are DPV profiles first and second scans (DES first black and second red dashed lines and VEN first black and second red straight lines), and the right and left panels are the DES and VEN molecular structures.

Anodic peaks, marked as 1a in Figure 2C, were observed at Ep_1*a*_ = 0.66 and 0.81 V for desvenlafaxine and venlafaxine, respectively. The maximum potential of DES related to the anodic process (Figure 2C dashed lines) is consistent with electroactive species containing a phenolic hydroxyl group.^27^ Since the study proposes potential oxidation pathways, a brief indication of the type of transformation, such as hydroxylation, would strengthen its goals. Alternatively, methylation of this electroactive group sterically hinders its oxidation, consistent with the observed current decay and positive potential shift seen for VEN in Figure 2B,C (straight lines).

The peak current intensities were also significantly affected by pH. Desvenlafaxine showed the highest analytical signal intensity at pH 7.0 (neutral), while venlafaxine had its highest signal intensity observed at pH 9.0 (alkaline). This suggests that venlafaxine has a lower proton dependence, which is consistent with the methylation of the hydroxyl group. In addition, a significant reduction in the anodic peak current in acidic pH was observed for both species.

Both medications presented similar cyclic voltammetric profiles at different scan rates, in which diffusional oxidation processes, marked as 1a in Figure 3A,B, were evidenced by the linearity between the intensity of the analytical signal versus the square root of the scan rate.^27,28^ The absence of cathodic peaks in the CV and SWV assays suggests that desvenlafaxine undergoes irreversible oxidation. This may indicate distinct ionized species or pH-dependent degradation byproducts for both compounds. The predominant transfer process, reversibility, and correlation with concentration were verified by means of the irreversibility of electrochemical oxidation.^21,22^

**Figure 3:**
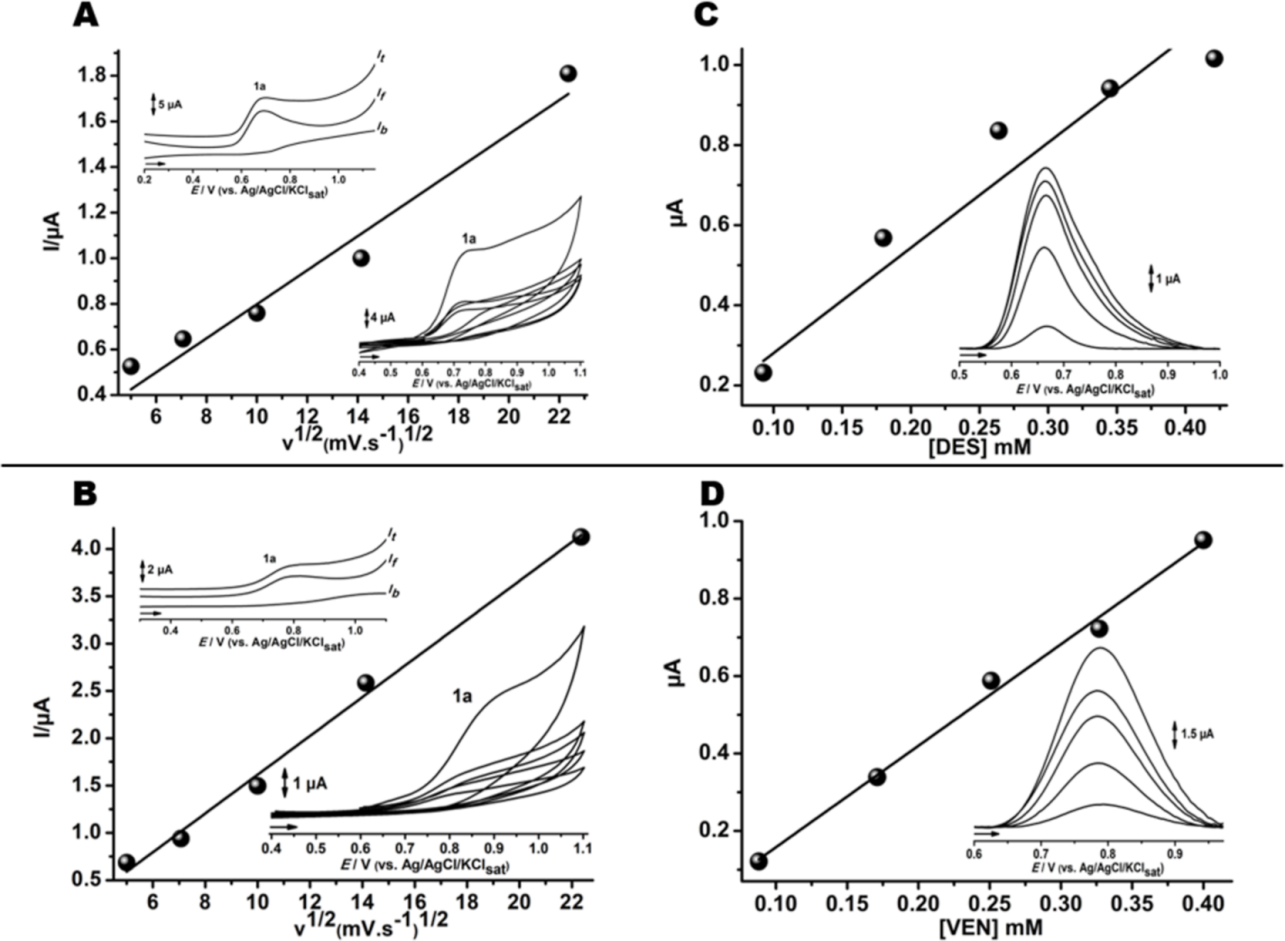
Cyclic voltammograms obtained at different scan rates (25, 50, 100, 250, 500 mV s^–1^) of DES (A) and VEN (B) (Inset: the respective SW voltammogramms). Calibration graphs increasing the concentrations of the analyte DES (C) and VEN (D). Inset: the related DP voltammograms. All voltammetric methods were performed with GCE electrode in pH 7.0 PBS.

The quantitative calibration curve to increase analyte concentration at pH 7.0 is shown in Figure 3C,D. A linear response was observed from 0.05 to 0.4 mM for DES and VEN with correlation coefficients of 0.93 and 0.95, respectively. Desvenlafaxine exhibits far stronger adsorptive behavior, as can be seen in the reduction in the analytical signal in the second scan (Figure 2C). This can be explained by the instability of the phenoxy intermediate, which undergoes an electropolymerization reaction, leading to an insulator film at the electrode surface. The degrease of the electroactive electrode area can result in lower correlation coefficient values for DES in relation to VEN species.

The molecular orbital energy levels of the antidepressants DES and VEN in both neutral and protonated forms, including their oxidation product, can be seen in Figure 4. Venlafaxine has a tertiary amine group with a p*K*_a_ of 9.24 and a phenolic hydroxyl group with a p*K*_a_ ranging from 10 to 12. The p*K*_a_ values of the amine and phenolic hydroxyl groups suggest that they are mainly in their protonated form under physiological pH conditions. Desvenlafaxine, a major active metabolite of venlafaxine, has p*K*_a_ values of 9.45 for the amine group and 10.66 for the phenolic hydroxyl group, reflecting similar ionization characteristics. These p*K*_a_ values suggest that both desvenlafaxine and venlafaxine can exist in neutral or protonated forms, depending on the pH of the solution. Consequently, both compounds were evaluated in neutral forms (desvenlafaxine Figure 4A and venlafaxine Figure 4C) and protonated forms (desvenlafaxine Figure 4B and venlafaxine Figure 4D), with the oxidation product denoted as Figure 4E.

**Figure 4:**
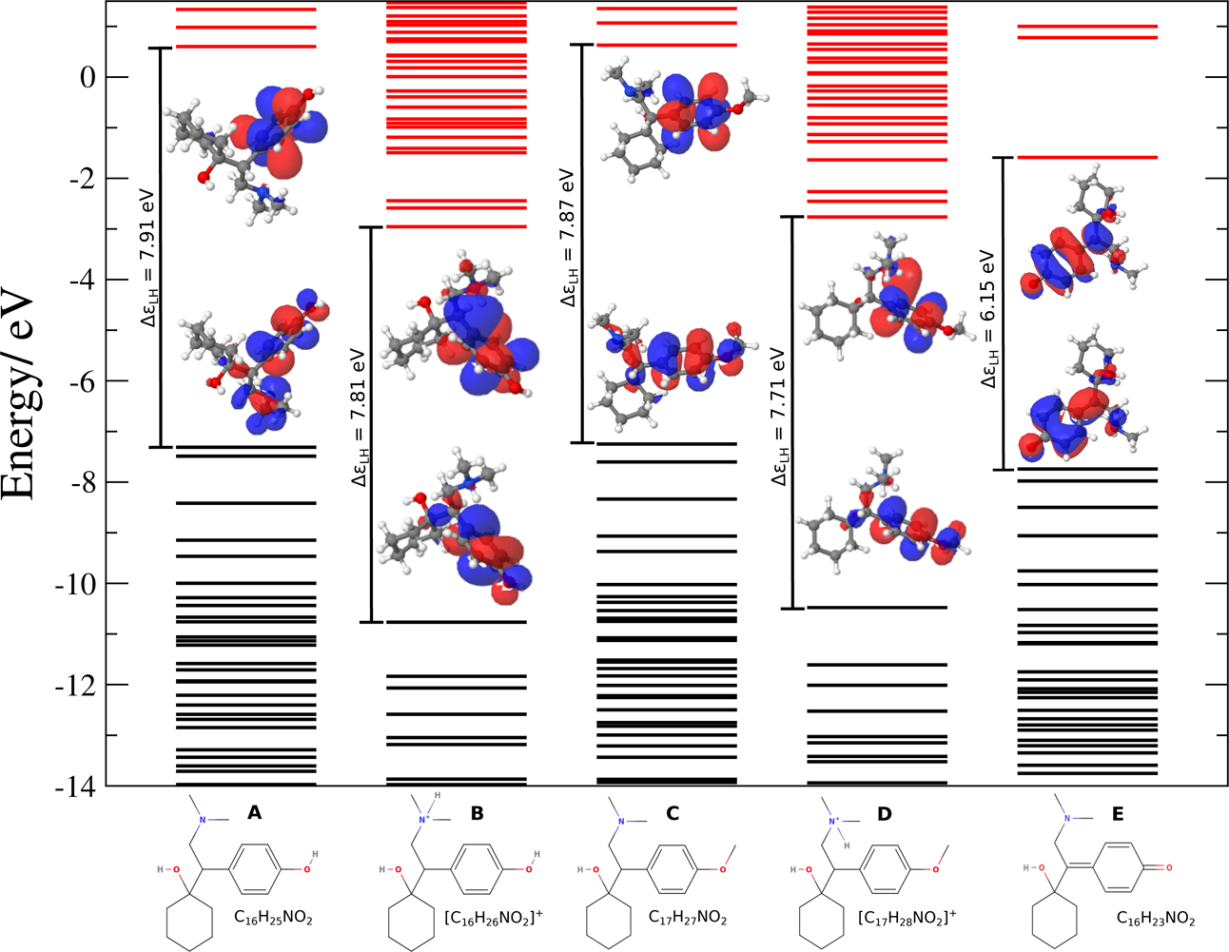
Molecular orbital energy levels of desvenlafaxine and venlafaxine in both neutral and protonated forms, and their oxidation product. The black and red energy level bars represent respectively the occupied and the unoccupied molecular orbitals (HOMO and LUMO, respectively). Δϵ_LH_ is the HOMO–LUMO gap in electronvolts units. (A) is the neutral desvenlafaxine, (B) the protonated desvenlafaxine, (C) the neutral venlafaxine, (D) the protonated venlafaxine and E the oxidation product.

The energy difference between the highest occupied molecular orbital (HOMO) and the lowest unoccupied molecular orbital (LUMO) is almost constant for all molecules, ranging from 7.71 to 7.91 eV, with the protonated species showing a smaller HOMO–LUMO gap for both molecular species. The most significant difference is observed in the oxidation product, which has a gap of 6.15 eV. The spatial distributions of the HOMO and LUMO of each molecular species studied are depicted in Figure 4, within the gap between the occupied and unoccupied energy levels, showing that the orbitals HOMO and LUMO are located in the aromatic ring of the molecules. Observing the differences in the energy levels of the HOMO between VEN and DES species, a significant difference of approximately 0.12 eV between the nonprotonated species and about 0.22 eV between the protonated species can be verified. This difference can influence the oxidation hardness of both species, potentially explaining the observed potential shift in their oxidation processes during electroanalytical tests.

On the basis of the p*K*_a_ values and analytical results, the free energies of reaction between the drugs and water or hydroxide ions were assessed, with the drugs considered in their neutral forms. The Gibbs free energy values calculated at the theoretical level wB97X-D3/def2-TZVP in the gas phase and in a solvent medium at wB97X-D3/def2-TZVP/SMD are summarized in Table 2. As shown, the solvent interaction alters the energetic profile of the reaction. The variation in Gibbs free energy in the gas phase indicates (Δ*G*_gas_) that interactions between desvenlafaxine and venlafaxine are energetically favorable with the hydroxide ion and unfavorable in water. In contrast, this profile is reversed in the solvent medium, indicating that there is no competition between the hydronium ions from the self-ionization of water and the water molecules, thus supporting the optimal results at pH 7. The distinct profiles between the gas and solvated phases can be attributed to the negative charge of the hydroxide ion, which enhances its interaction in the gas phase. Furthermore, in a solvent medium, the hydronium ion is solvated by water molecules, thereby decreasing its availability and making the interaction between the drug and the hydronium ion thermodynamically unfavorable, decreasing the overpotential of oxidation. It is important to note that the chemical reactions evaluated occur in a heterogeneous medium and involve complex mechanisms. Consequently, the thermodynamic analysis conducted in this study does not account for the full complexity of the reaction mechanisms or the kinetics of each step. However, experimental studies^27,29^ suggest that the oxidation process leads to the formation of carbocation in the aromatic ring, followed by a demethylation step. Given the structural similarity between venlafaxine and desvenlafaxine, it is reasonable to expect that the free energy for the carbocation formation and the demethylation process would be similar for both compounds. This suggests that the values of (Δ*G*_gas_) and (Δ*G*_sol_) obtained in Table 2 are consistent with the experimental observations.

**Table 2:** Gibbs Free Energy in kcal/mol Units Calculated for the Reaction between the DES and VEN with Species H_2_O and OH^–^ ^a^

reactants	products	Δ*G*_gas_	Δ*G*_sol_
DES + 2H_2_O	2H_3_O^+^	439.22	–175.00
DES + 2OH^–^	H_2_O	–32.92	189.13
VEN + 2H_2_O	2H_3_O^+^ + CH_3_OH	435.41	–177.00
VEN + 2OH^–^	H_2_O + CH_3_OH	–36.73	187.20

a The ketone is omitted in the products but take into account in the calculations.

The wavelength and oscillator strength parameters, in addition to the main orbital contributions for the first three singlet-to-singlet electronic transitions, are reported in Table 3. These results are generally compared with the UV–vis spectroscopic data. The results show that the first three electronic excitations occur in the UV region, with the highest intensities observed in the UVB range. The lowest-energy transitions corresponding to the longest wavelengths have small oscillator strengths for all molecules. The HOMO–LUMO transitions are weak for almost all compounds, except the oxidation product, which has an oscillator strength of 0.7121 almost three times the highest value found for protonated venlafaxine. The HOMO → LUMO+1 transition exhibits the highest absorption intensity for the neutral species of desvenlafaxine and venlafaxine. In contrast, for ionized desvenlafaxine and venlafaxine, the HOMO → LUMO+2 transition becomes the highest absorption peak.

**Table 3:** TD–DFT Wavelength (λ), Oscillator Strength (*f*) in Arbitrary Units, and the Main Orbital Contribution for the First Three Electronic Transitions

molecules		λ/nm	*f*	main transition
C_16_H_25_NO_2_	first	234.99	0.0253	HOMO–1 → LUMO
	second	213.04	0.0028	HOMO → LUMO
	third	202.68	0.1073	HOMO → LUMO+1
[C_16_H_26_NO_2_]^+^	first	235.93	0.0262	HOMO → LUMO
	second	204.69	0.0896	HOMO → LUMO+2
	third	199.37	0.0333	HOMO → LUMO+1
C_17_H_27_NO_2_	first	238.68	0.0227	HOMO → LUMO
	second	208.96	0.2290	HOMO → LUMO+1
	third	196.01	0.0291	HOMO–1 → LUMO
[C_17_H_28_NO_2_]^+^	first	240.32	0.0211	HOMO → LUMO+1
	second	219.93	0.0193	HOMO → LUMO
	third	213.25	0.2477	HOMO → LUMO+1
C_16_H_23_NO_2_	first	389.88	0.0003	HOMO–2 → LUMO
	second	301.54	0.0169	HOMO–1 → LUMO
	third	282.21	0.7121	HOMO → LUMO

The electrochemical reaction occurs through the transfer of electrons between defined molecular orbitals. In this process, an electron is transferred from a high energy orbital, such as the highest occupied molecular orbitals (HOMO, HOMO–1), associated with the reducing agent—specifically, the VEN e DES analysis in the present study—to a lower energy vacant molecular orbital, such as the lowest unoccupied molecular orbital (LUMO, LUMO+1), associated with the oxidizing agent. According to Figure 1, the orbitals HOMO and HOMO–1 in the neutral forms of both drugs are located in the aromatic ring and exhibit comparable energy levels, approximately 7.4 eV. In the protonated forms, these orbitals remain centered on the aromatic ring but are more than 3 eV more stable than in the deprotonated forms. This indicates that oxidation of both drugs probably occurs through the transfer of an electron from the orbital HOMO or HOMO–1 of the deprotonated molecules, with the resultant positive charge localized on the aromatic ring. In this context, it can be inferred that the potential shift observed in the oxidation processes of the acetylenes is attributable both to the difference in energy between the orbitals and to the presence of the methyl group in venlafaxine, which contributes to the stabilization of the positive charge developed on the aromatic ring. The potential similarity between the oxidation mechanisms of VEN and DES is further corroborated by the reaction’s closely comparable free-energy values, both in the gas phase and in solution. These findings also support the conclusion that the demethylation process in venlafaxine occurs after the oxidation process and is facilitated by the presence of a water molecule.

A scheme of the venlafaxine oxidation pathway is proposed for the desvenlafaxine molecule in Figure 5A, and two alternative pathways for venlafaxine are presented in Figure 5B,C. The alternative oxidation pathway of Figure 5C is very similar to that proposed by Sanghavi and Srivastava^27^ The energy levels for each step of the process were computed in DFT/def-TZVP/M06-2x and are given in hartree units. The energy levels drawn in black states are the energy of the optimized molecular structures (stable structures) presenting only real numbers for the vibrational frequencies, whereas the energy levels drawn in red are molecular energies correspondent to the diabatic ionization or elimination process disregarding the nuclear motion. For the venlafaxine molecule, in both mechanism B and C, the energies were adjusted by adding the energy of the methyl cation (−39.4664 hartree) to the products after elimination of the methyl group. The energy of the CH_3_^+^ ion used to shift the energies was computed at the same level of theory. For both molecules, the single occupied molecular orbital, in the first step of the oxidation pathway, is distributed throughout the aromatic ring. Despite that, to facilitate the picture of the oxidation process, the charge was placed in the carbon atom with the highest charge from the Mulliken load values from the calculations. The comparison of the proposed oxidation mechanics for VEN in Figure 5B,C is the same in the first stage of the process, and they are different in the second and third steps. In the second stage, the energy required for elimination to occur is greater in B than in C. The energy difference between the stable geometric states in B has a value of 0.6436 hartree due to the oxidation and elimination of the proton (H^+^), while in C it has a value of 0.2646 hartree corresponds to the elimination of CH_3_^+^. In the third stage, the overall process is inverted in proposals B and C in relation to the preceding step. Elimination of CH_3_^+^ in B has a value of 0.7013 hartree and oxidation and elimination of (H^+^) with a value of 0.2291 hartree. Therefore, in terms of total energy to reach the oxidation product, mechanism B has a lower energy difference (0.8727 hartree) than mechanism C (0.9659 hartree).

**Figure 5:**
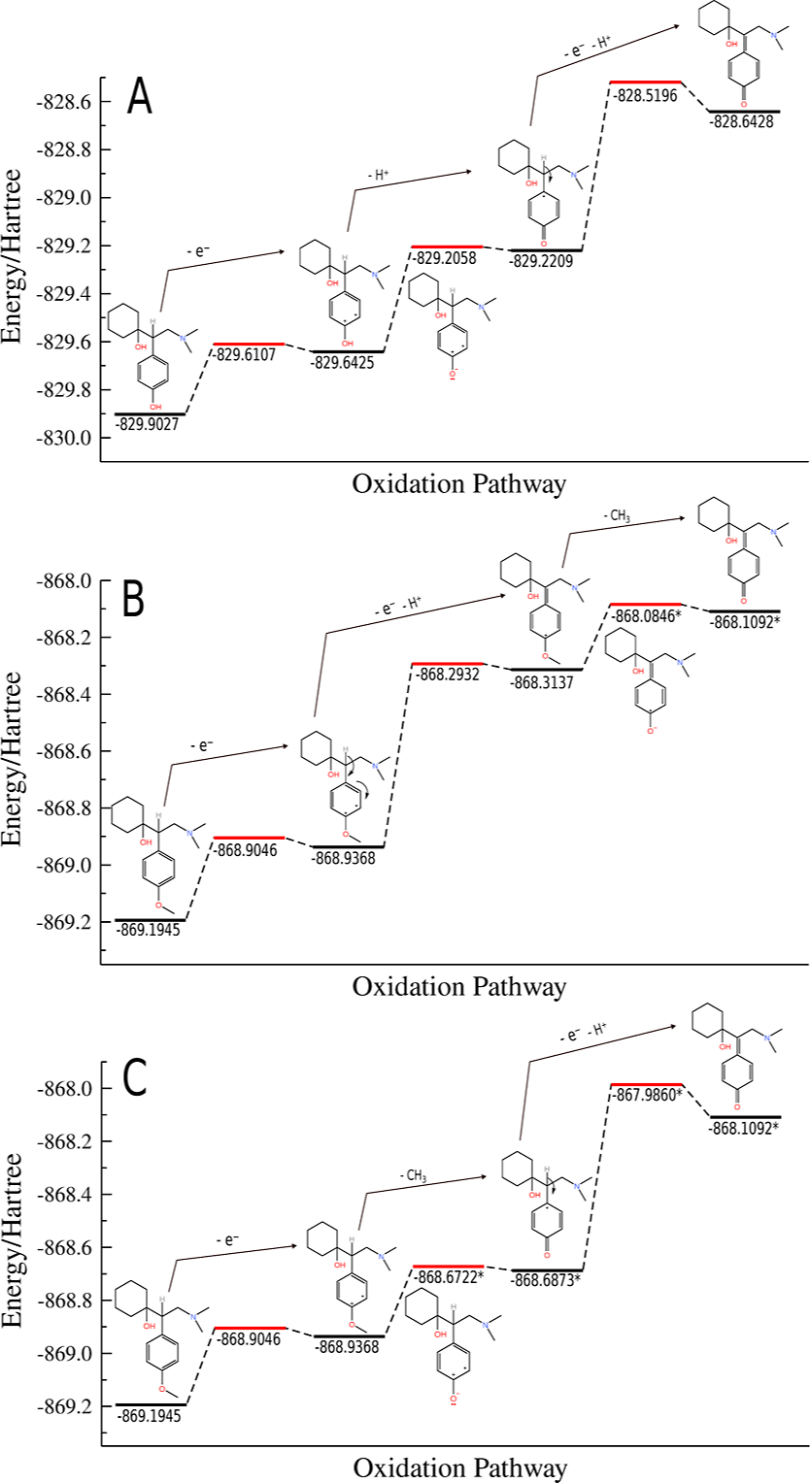
Mechanistic proposal oxidation pathways for DES (A) and VEN (B) and (C). The energy states drawn in black are stable molecular geometries representing a minimum in the potential energy surface. The red levels are diabatic ionization or elimination states disregarding the nuclear motion. The energy levels marked with * where shift by the CH_3_^+^ electronic energy value.

The integrated analysis of the quantum descriptors (Table 4) allows inferences about not only the relative stability of the species but also their global reactivity and the preferred directionality of electron-transfer processes. Specifically, systems characterized by high global hardness and less positive chemical potentials demonstrate greater thermodynamic stability, while high values of ω indicate a pronounced electrophilic character, suggesting a propensity to act as charge acceptors in chemical interactions. This approach thus provides a robust theoretical basis for predicting and rationalizing reactive behaviors in molecular chemistry and advanced computational chemistry.

**Table 4:** Calculated Electronic Descriptors for Venlafaxine, Desvenlafaxine, and Their Common Oxidation Product^a^

molecule	*I* = −ϵ_HOMO_	*A* = −ϵ_LUMO_	μ	η	ω
Desvenlafaxine	8.182	–1.662	–3.2597	4.9220	1.0794
Venlafaxine	7.756	–2.021	–2.8688	4.888	0.8410
Oxidation Product	8.641	0.609	–4.6251	4.0156	–2.6636

a All values are given in electronvolts (eV).

The lower ionization energy *I* of VEN (7.756 eV) compared to DES (8.182 eV) indicates a greater thermodynamic facility for electron removal, consistent with the more anodic oxidation potential observed experimentally for VEN. This lower *I* value for VEN is intrinsically linked to the methyl group on the aromatic ring, which, through an electron-donating effect, raises the HOMO energy, making the system more susceptible to oxidation. Paradoxically, this same effect stabilizes the radical cation formed initially, requiring a higher overpotential to overcome the kinetic barrier associated with the subsequent demethylation step, as detailed in the mechanisms in Figure 5B,C. The Chemical Potential μ, which measures the tendency of a system to lose electron density, is less negative for VEN (−2.869 eV) than for DES (−3.260 eV). This confirms the greater thermodynamic tendency of VEN to act as a reducing agent (electron donor), despite the kinetic barriers. Following oxidation, the formed product exhibits a significantly more negative chemical potential (−4.625 eV), indicating substantial electronic stabilization, which acts as the driving force for the reaction. The Global Hardness (η), related to the resistance to changes in electron distribution, is slightly lower for VEN (4.888 eV) relative to DES (4.922 eV). A lower hardness implies greater polarizability and consequently higher intrinsic reactivity, which is consistent with the reactive profile of VEN. The oxidation product exhibits an even further reduced global hardness (4.016 eV), reflecting its pronounced electrophilic character. The Electrophilicity Index (ω) of the oxidation product (2.664 eV) is notably higher than that of the precursors (VEN: 0.841 eV; DES: 1.079 eV). This drastic increase confirms that the oxidation generates a highly electrophilic species that can readily undergo nucleophilic attacks, such as the hydration proposed in the mechanism, or participate in electropolymerization reactions, as suggested by the decay of the analytical signal in the second voltammetric scan for DES.

## 4 Conclusions

The investigation revealed that VEN and DES exhibit distinct pH-dependent electrochemical behaviors, with DES showing higher anodic peak intensities at neutral pH, while VEN peaks at alkaline pH. These observations suggest variations in their proton dependence as a result of structural differences, which was further confirmed by the absence of cathodic peaks indicating the irreversible nature of their oxidation processes. Computational insights from DFT calculations provided a deeper understanding of the molecular orbital profiles, indicating that the primary sites for oxidation are the aromatic rings. The Gibbs free energy variations in different solvent environments highlighted the significant role of the solvent in modulating the energetic profiles of interactions between the drugs and ions. A comprehensive oxidation mechanism with calculations of the intermediate stable structures is drawn. The proposed oxidation mechanism identified the stereogenic center as the primary oxidation site for VEN and the hydroxyl group on the aromatic ring for DES. This study provides valuable insights into the redox behavior of VEN and its metabolite DES through comprehensive electroanalytical and computational analyses. The combination of electroanalytical techniques with computational chemistry has shown to be a promising approach for elucidating the redox behavior of complex molecules, improving our understanding of the electrochemical properties of these antidepressants, and paving the way for the development of more effective therapeutic agents with optimized redox properties.

## Supplementary Material





## Notes

The Supporting Information includes: Figures S1–S4 the comparison between theoretical and experimental molecular geometries for distinct spatial orientations, Figures S5–S7 the spatial distribution for a few internal molecular orbitals, Tables S1–S4 the TD-DFT molecular orbital contributions to the ten first electronic transitions, and Listing 1–4 the Cartesian coordinates (xyz) of the optimized molecular geometries at the DFT/M06–2X/def2-TZVP level for VEN and DES (PDF)
The raw electrochemical data, including cyclic voltammetry (CV), square-wave voltammetry (SWV), and differential pulse voltammetry (DPV) of concentration-dependent studies for both molecules (XLSX)
