# Supplementary material for: Electronic Structure and Redox of the Antidepressants Venlafaxine and Desvenlafaxine
Source: ACS Omega. 2025 Nov 25;10(48):59290–9. doi: 10.1021/acsomega.5c08632 (PMC12771420; doi:10.1021/acsomega.5c08632)
Supplement: Supplementary file 1 [file ao5c08632_si_001.pdf]

Supporting Information

Electronic Structure and Redox of the Antidepressants Venlafaxine and Desvenlafaxine

Jhon Kennedy Alves Pereira,[†] Eufrásia de Sousa Pereira,[‡] Bárbara Júlia Gonçalves Dutra,[¶] Isaac Yves Lopes de Macêdo,[†] Arthur Saldanha Guimarães,[†] Bruno Junior Neves,[‡] Eric de Souza Gil,[*,‡] and Freddy Fernandes Guimarães[*,¶]

†*Laboratory of Pharmaceutical and Environmental Analysis, Faculdade de Farmácia, Universidade Federal de Goiás, 74605-170, Goiás, Brazil.*

‡*Laboratory of Cheminformatics, Faculdade de Farmácia, Universidade Federal de Goiás, Goiânia, 74605-170, Goiás, Brazil.*

¶*Institute of Chemistry, Universidade Federal de Goiás, Goiânia, 74605-170, Goiás, Brazil.*

E-mail: ericsgil@ufg.br; freddy@ufg.br

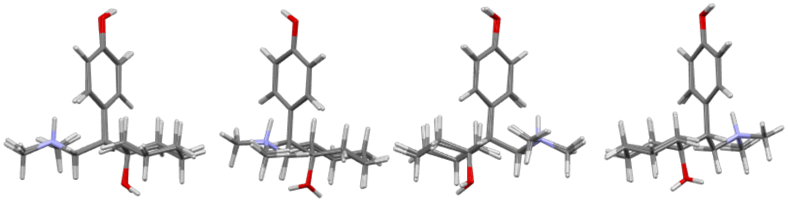

Figure S1: Superposition of the neutral $C_{16}H_{26}NO_2$ and protonated $[C_{16}H_{27}NO_2^+]$ forms of desvenlafaxine molecule, depicting distinct molecular orientations.

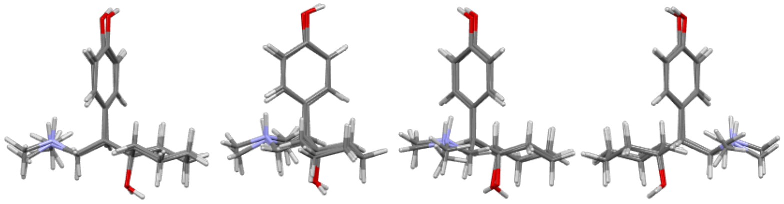

Figure S2: Superposition of the experimental and DFT-optimized crystallographic structures of desvenlafaxine $C_{16}H_{26}NO_2$ molecule, depicting distinct molecular orientations.

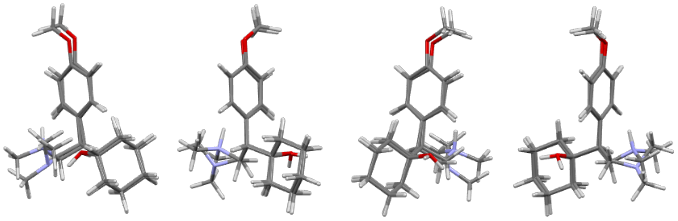

Figure S3: Superposition of the neutral $C_{17}H_{27}NO_2$ and protonated $[C_{17}H_{28}NO_2^+]$ forms of venlafaxine molecule, depicting distinct molecular orientations.

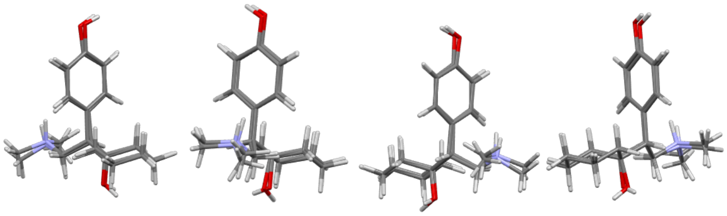

Figure S4: Superposition of the experimental and DFT-optimized crystallographic structures of venlafaxine $C_{17}H_{27}NO_2$ molecule, depicting distinct molecular orientations.

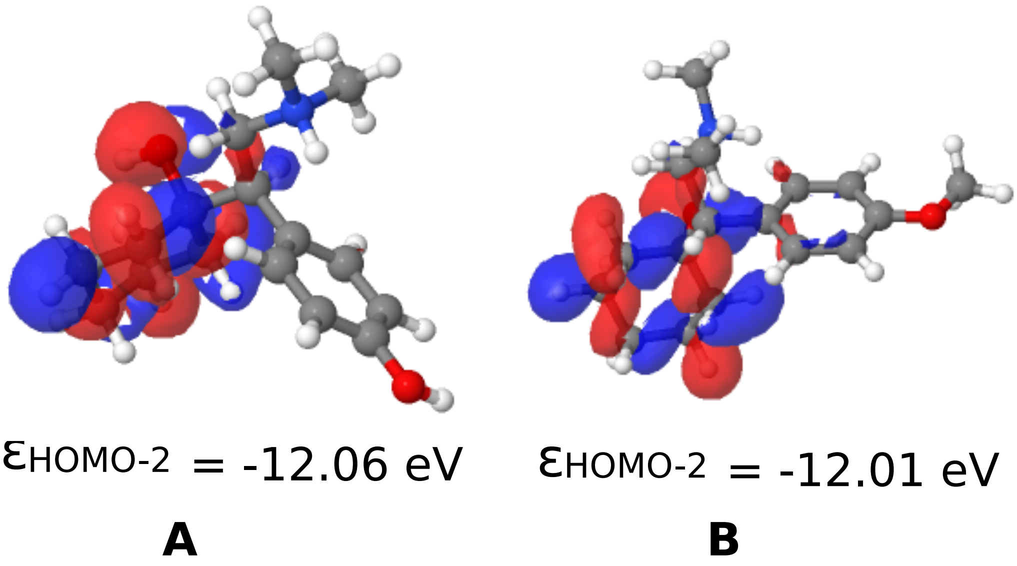

Figure S5: Spatial distribution of the HOMO−2 orbital for the neutral species. The orbital is localized on the cyclohexane moiety. Panels show (A) desvenlafaxine and (B) venlafaxine.

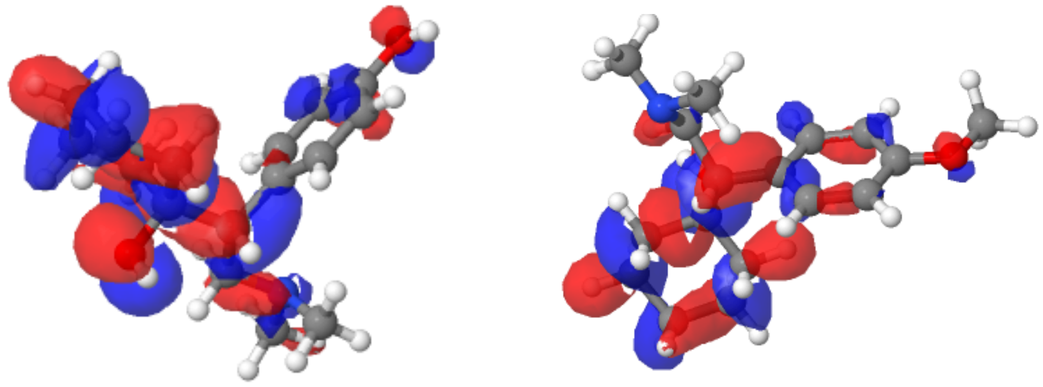

$\varepsilon_{HOMO\text{-}3}$ = -9.14 eV $\varepsilon_{HOMO\text{-}3}$ = -9.06 eV

A **B**

Figure S6: Spatial distribution of the HOMO−3 orbital for the protonated species. The orbital is localized on the cyclohexane moiety. Panels show (A) desvenlafaxine and (B) venlafaxine.

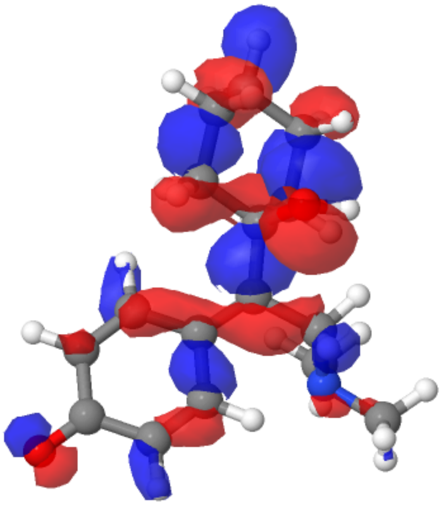

Figure S7: Spatial distribution of the HOMO−4 orbital for the oxidation product. The orbital is localized on the cyclohexane moiety.

Table S1: TD-DFT calculated energy, wavelengths λ, oscillator strengths f in arbitrary units, and molecular orbital contributions to the first ten electronic transitions of $C_{16}H_{26}NO_2$. Orbitals 72 and 73 are HOMO and LUMO of $C_{16}H_{26}NO_2$ molecule, respectively.

**Excited State**	**Energy/eV**	**λ/nm**	f	**Transition**	**Coefficient**
1	5.2762	234.99	0.0253	70 → 74	0.28675
				71 → 73	0.57520
				72 → 73	0.27321
2	5.8197	213.04	0.0028	71 → 73	-0.24874
				72 → 73	0.63516
				72 → 74	-0.12417
3	6.1171	202.68	0.1073	70 → 73	-0.25936
				71 → 73	-0.12997
				71 → 74	0.38672
				72 → 73	0.11113
				72 → 74	0.49130
4	6.2692	197.77	0.0072	70 → 73	-0.22624
				71 → 74	0.45581
				72 → 74	-0.44099
				72 → 75	-0.16244
5	6.4348	192.68	0.0139	72 → 74	-0.18867
				72 → 75	0.49080
				72 → 77	0.23666
				72 → 78	0.29788
				72 → 80	0.12740
				72 → 87	0.12112

Table S1: TD-DFT calculated energy, wavelengths λ, oscillator strengths f in arbitrary units, and molecular orbital contributions to the first ten electronic transitions of $C_{16}H_{26}NO_2$ (continuation).

Excited State	Energy/eV	λ/nm	f	Transition	Coefficient
6	6.9526	178.33	0.1412	70 $\rightarrow$ 73	0.23996
				70 $\rightarrow$ 74	-0.10078
				71 $\rightarrow$ 74	0.14751
				71 $\rightarrow$ 75	0.26347
				71 $\rightarrow$ 76	0.48972
				72 $\rightarrow$ 75	0.11376
				72 $\rightarrow$ 76	0.19262
7	6.9940	177.27	0.6238	70 $\rightarrow$ 73	0.53753
				71 $\rightarrow$ 74	0.30003
				71 $\rightarrow$ 76	-0.21560
				72 $\rightarrow$ 75	-0.12333
				72 $\rightarrow$ 78	0.13796
8	7.0193	176.63	0.0311	70 $\rightarrow$ 73	-0.10962
				71 $\rightarrow$ 76	0.20528
				72 $\rightarrow$ 75	-0.33081
				72 $\rightarrow$ 78	0.45255
				72 $\rightarrow$ 82	0.15690
				72 $\rightarrow$ 87	0.15848
9	7.1142	174.28	0.2689	70 $\rightarrow$ 74	0.60285
				71 $\rightarrow$ 73	-0.27488
				71 $\rightarrow$ 76	0.12382

Table S1: TD-DFT calculated energy, wavelengths λ, oscillator strengths f in arbitrary units, and molecular orbital contributions to the first ten electronic transitions of $C_{16}H_{26}NO_2$ (continuation).

**Excited State**	**Energy/eV**	**λ/nm**	f	**Transition**	**Coefficient**
10	7.1679	172.97	0.0420	69 → 75	0.14191
				70 → 74	0.14312
				71 → 75	0.57115
				71 → 76	-0.20920
				71 → 79	0.15256

Table S2: TD-DFT calculated energy, wavelengths λ, oscillator strengths f in arbitrary units, and molecular orbital contributions to the first ten electronic transitions of $[C_{16}H_{26}NO_2]^+$. Orbitals 72 and 73 are HOMO and LUMO of $[C_{16}H_{26}NO_2]^+$ cation, respectively.

**Excited State**	**Energy/eV**	**λ/nm**	f	**Transition**	**Coefficient**
1	5.2552	235.93	0.0262	71 → 75	0.28715
				72 → 73	0.56598
				72 → 74	-0.28969
2	6.0572	204.69	0.0896	71 → 73	-0.31559
				71 → 74	0.14336
				72 → 73	0.15088
				72 → 74	0.13037
				72 → 75	0.57511
3	6.2188	199.37	0.0334	72 → 73	0.31174
				72 → 74	0.56533
				72 → 75	-0.19784
				72 → 77	0.10503

Table S2: TD-DFT calculated energy, wavelengths λ, oscillator strengths f in arbitrary units, and molecular orbital contributions to the first ten electronic transitions of $[C_{16}H_{26}NO_2]^+$(continuation).

**Excited State**	**Energy/eV**	**λ/nm**	f	**Transition**	**Coefficient**
4	6.8022	182.27	0.4443	71 → 73	0.57743
				71 → 74	0.11723
				71 → 75	0.17749
				72 → 74	0.12257
				72 → 75	0.28507
5	6.9960	177.22	0.4690	71 → 73	-0.20406
				71 → 74	0.38459
				71 → 75	0.46067
				72 → 73	-0.17928
				72 → 74	0.11876
				72 → 75	-0.16218
6	7.1329	173.82	0.1042	71 → 74	0.50156
				71 → 75	-0.33789
				72 → 73	0.11372
				72 → 74	-0.14061
				72 → 75	-0.10698
				72 → 76	-0.20459
7	7.3144	169.51	0.0627	71 → 74	0.13857
				71 → 75	-0.18549
				72 → 76	0.62116

Table S2: TD-DFT calculated energy, wavelengths λ, oscillator strengths f in arbitrary units, and molecular orbital contributions to the first ten electronic transitions of $[C_{16}H_{26}NO_2]^+$(continuation).

**Excited State**	**Energy/eV**	**λ/nm**	f	**Transition**	**Coefficient**
8	7.5883	163.39	0.0143	69 → 73	0.14998
				72 → 77	0.50047
				72 → 78	0.25364
				72 → 80	-0.10542
				72 → 81	0.21310
				72 → 82	-0.18056
9	7.7217	160.57	0.0023	64 → 73	0.13462
				65 → 73	0.21223
				65 → 74	-0.10828
				69 → 73	0.44527
				69 → 74	-0.12138
				70 → 73	0.30502
				72 → 77	-0.14387
				72 → 78	0.10143
				72 → 81	-0.10604
10	7.8271	158.40	0.0004	65 → 73	-0.17800
				69 → 73	0.11941
				70 → 73	-0.20203
				72 → 77	-0.24952
				72 → 78	0.41945
				72 → 79	-0.30278

Table S3: TD-DFT calculated energy, wavelengths λ, oscillator strengths f in arbitrary units, and molecular orbital contributions to the first ten electronic transitions of $C_{17}H_{27}NO_2$. Orbitals 76 and 77 are HOMO and LUMO of $C_{17}H_{27}NO_2$ molecule, respectively.

**Excited State**	**Energy/eV**	λ**/nm**	f	**Transition**	**Coefficient**
1	5.1946	238.68	0.0227	74 → 78	0.29662
				75 → 77	-0.27794
				76 → 77	0.56940
2	5.9333	208.96	0.2290	74 → 77	-0.27155
				75 → 78	-0.25129
				76 → 78	0.58984
3	6.3253	196.01	0.0291	75 → 77	0.61578
				76 → 77	0.31186
4	6.4450	192.37	0.0130	75 → 78	0.41365
				75 → 79	0.20570
				75 → 80	-0.21797
				75 → 81	0.14266
				75 → 82	0.16807
				75 → 83	-0.16188
				75 → 88	-0.10660
				76 → 78	0.19422
				76 → 79	0.12222
				76 → 80	-0.12413

Table S3: TD-DFT calculated energy, wavelengths λ, oscillator strengths f in arbitrary units, and molecular orbital contributions to the first ten electronic transitions of $C_{17}H_{27}NO_2$ (continuation).

Excited State	Energy/eV	λ/nm	f	Transition	Coefficient
5	6.6595	186.18	0.0202	74 → 78	0.11451
				75 → 78	0.43286
				75 → 79	-0.27608
				75 → 80	0.17363
				75 → 81	-0.13003
				75 → 82	-0.16222
				76 → 78	0.23511
				76 → 79	-0.18964
6	6.8241	181.69	0.7493	74 → 77	0.61320
				75 → 78	-0.22039
				76 → 78	0.18450
7	6.9046	179.57	0.2358	74 → 77	-0.11523
				74 → 78	0.60332
				75 → 77	0.14195
				76 → 77	-0.24537
8	6.9729	177.81	0.0194	75 → 79	0.23761
				75 → 81	-0.14290
				75 → 83	0.22599
				75 → 88	0.17726
				76 → 79	0.43542
				76 → 83	0.16786

Table S3: TD-DFT calculated energy, wavelengths λ, oscillator strengths f in arbitrary units, and molecular orbital contributions to the first ten electronic transitions of $C_{17}H_{27}NO_2$ (continuation).

**Excited State**	**Energy/eV**	**λ/nm**	f	**Transition**	**Coefficient**
9	7.0404	176.11	0.0015	75 → 79	0.40050
				75 → 81	0.11700
				75 → 83	0.12962
				76 → 79	-0.27614
				76 → 80	-0.21317
				76 → 81	-0.32055
				76 → 85	-0.11674
10	7.2718	170.50	0.0186	73 → 79	-0.10350
				75 → 79	0.19923
				75 → 81	-0.16601
				75 → 83	0.14475
				76 → 79	-0.28180
				76 → 81	0.33489
				76 → 82	-0.22627
				76 → 83	-0.21638
				76 → 85	0.16203
				76 → 88	0.10487

Table S4: TD-DFT calculated energy, wavelengths λ, oscillator strengths f in arbitrary units, and molecular orbital contributions to the first ten electronic transitions of $[C_{17}H_{28}NO_2]^+$. Orbitals 76 and 77 are HOMO and LUMO of $[C_{17}H_{28}NO_2]^+$ cation, respectively.

**Excited State**	**Energy/eV**	**λ/nm**	f	**Transition**	**Coefficient**
1	5.1591	240.32	0.0211	75 → 79	0.26443
				75 → 81	-0.11020
				76 → 78	0.62417
				76 → 81	-0.10450
2	5.6374	219.93	0.0193	76 → 77	0.68728
3	5.8140	213.25	0.2477	75 → 78	-0.24494
				76 → 79	0.62302
				76 → 81	-0.16222
4	6.4534	192.12	0.0201	75 → 78	0.18171
				75 → 79	-0.16276
				76 → 78	0.19227
				76 → 79	0.20384
				76 → 80	0.34391
				76 → 81	0.45102
5	6.6581	186.21	0.0041	75 → 77	0.69360
6	6.7365	184.05	0.4897	75 → 78	0.45581
				75 → 79	-0.22729
				76 → 79	0.14055
				76 → 80	-0.40274
				76 → 83	-0.10991

Table S4: TD-DFT calculated energy, wavelengths λ, oscillator strengths f in arbitrary units, and molecular orbital contributions to the first ten electronic transitions of $[C_{17}H_{28}NO_2]^+$ (continuation).

Excited State	Energy/eV	λ/nm	f	Transition	Coefficient
7	6.7632	183.32	0.3177	75 → 78	0.35553
				75 → 79	0.46578
				75 → 81	-0.18829
				76 → 78	-0.23577
				76 → 79	0.14025
				76 → 80	0.13013
8	6.8699	180.47	0.1262	75 → 78	-0.21268
				75 → 79	0.22859
				76 → 80	-0.34818
				76 → 81	0.46341
				76 → 83	-0.14737
9	7.0482	175.91	0.0039	74 → 77	-0.10449
				76 → 80	-0.15830
				76 → 82	0.48941
				76 → 83	0.32117
				76 → 84	-0.22718
				76 → 85	0.10715
				76 → 88	0.11180
10	7.1706	172.91	0.0124	73 → 77	-0.11156
				74 → 77	0.65516

Listing 1: $C_{16}H_{25}NO_2$ cartesian coordinates (xyz) optimized at DFT/M062-X/def2-TZVP

C	3.877047	−2.833883	11.725938
C	5.107359	−3.743890	11.604539
C	6.356100	−2.876254	11.459118
C	6.230307	−1.896009	10.301097
C	4.998621	−1.004500	10.447002
C	3.761133	−1.862408	10.580303
O	5.014436	−4.489886	10.377620
C	5.176187	−4.759672	12.798658
C	6.013848	−5.899570	12.503982
N	5.941896	−7.005283	13.548753
C	4.643755	−7.636974	13.818168
C	6.883130	−8.018601	13.116646
C	5.371874	−4.078821	14.147516
C	4.297674	−3.771265	14.953710
C	4.455310	−3.146621	16.168303
C	5.711826	−2.799283	16.617704
C	6.793652	−3.112310	15.847808
C	6.636631	−3.749945	14.627778
O	5.928254	−2.166441	17.820464
H	5.209217	−1.945925	18.146387
H	4.234462	−4.707653	10.248203
H	3.440809	−3.993125	14.668292
H	3.708659	−2.957423	16.690235
H	7.647361	−2.893353	16.144993
H	7.387733	−3.962801	14.121927

H	4.271401	−5.132430	12.847744
H	6.501011	−2.382438	12.281910
H	7.127507	−3.446397	11.312977
H	6.172307	−2.388471	9.467820
H	7.024340	−1.339485	10.264991
H	5.092074	−0.443083	11.232642
H	4.916122	−0.429519	9.670736
H	2.989280	−1.292520	10.720649
H	3.623021	−2.355107	9.756403
H	3.077675	−3.384057	11.758687
H	3.929927	−2.337791	12.558478
H	6.933802	−5.600690	12.429881
H	5.752948	−6.265967	11.644279
H	6.437612	−8.643666	12.539647
H	7.230116	−8.481117	13.883088
H	7.604899	−7.602388	12.640010
H	4.187258	−7.147436	14.507845
H	4.781251	−8.542318	14.105558
H	4.113374	−7.630629	13.017894

Listing 2: [$C_{16}H_{26}NO_2{}^{+}$] cartesian coordinates (xyz) optimized at DFT/M062-X/def2-TZVP

O	3.790820	−2.750030	11.987002
H	4.505100	−3.395810	12.010601
O	6.133040	4.681190	11.512599
H	6.374729	5.220930	10.753299
N	4.465064	2.659870	14.648402

H	4.076274	1.752940	14.363302
C	5.122101	1.174280	12.281800
C	6.028951	0.119884	12.351699
H	7.085531	0.333561	12.472997
C	5.617350	−1.200040	12.249599
H	6.344820	−2.001720	12.297898
C	4.267390	−1.493820	12.086301
C	3.339880	−0.455563	12.024303
H	2.294720	−0.698575	11.888304
C	3.768810	0.856047	12.118102
H	3.028820	1.645880	12.033503
C	5.604031	2.602500	12.408300
H	6.617721	2.583670	12.818498
C	5.759079	3.376650	11.071200
C	4.476328	3.441950	10.235202
H	4.154957	2.423820	10.001902
H	3.677759	3.923610	10.805303
C	4.697736	4.193650	8.921191
H	4.934596	5.243790	9.123761
H	3.768555	4.202760	8.350753
C	5.820005	3.556570	8.107300
H	5.520754	2.544620	7.816110
H	5.985253	4.115711	7.185940
C	7.107686	3.481920	8.922368
H	7.891915	2.977450	8.357347
H	7.485176	4.493580	9.110707
C	6.883548	2.747330	10.243598

H	7.799019	2.743120	10.840997
H	6.619617	1.707000	10.031998
C	4.732912	3.410450	13.371201
H	3.756362	3.631440	12.942903
H	5.211083	4.348760	13.643801
C	3.460385	3.367889	15.485803
H	3.872026	4.334129	15.769403
H	3.253296	2.774429	16.372703
H	2.551904	3.506350	14.905504
C	5.710935	2.383719	15.415700
H	6.344464	1.720809	14.833899
H	5.444786	1.909339	16.356700
H	6.214395	3.330799	15.598999

Listing 3: $C_{17}H_{27}NO_2$ cartesian coordinates (xyz) optimized at DFT/M062-X/def2-TZVP

C	4.678703	−0.734123	4.253031
C	3.132383	−1.050575	4.170986
C	2.535974	−0.693868	5.588747
C	2.808776	0.711944	6.059737
C	4.318156	1.012973	6.084083
C	4.950590	0.632142	4.717629
O	2.096171	1.554662	5.074780
C	2.192408	1.054140	7.525378
C	0.804190	0.471686	7.715033
C	−0.406941	0.991346	7.203448
C	−1.649430	0.389865	7.374542

C	−1.754929	−0.709285	8.156656
C	−0.544888	−1.276317	8.602357
C	0.667451	−0.692040	8.439644
O	−3.035655	−1.146683	8.298718
C	−3.272499	−2.349360	8.915534
C	2.256013	2.537489	7.803733
N	2.749655	2.973549	9.233526
C	1.795898	2.466446	10.304252
C	2.968385	4.378730	9.417692
H	4.741456	0.508731	6.792930
H	4.459178	1.958208	6.256141
H	2.203426	2.345837	5.265701
H	−0.371978	1.784004	6.718931
H	−2.401935	0.741900	6.953187
H	2.761460	0.622926	8.165774
H	−0.580867	−2.100360	9.032748
H	1.369493	2.906735	7.667810
H	2.843159	2.941205	7.147576
H	2.122204	4.827480	9.469430
H	3.467422	4.723348	8.673794
H	3.459891	4.522904	10.228209
H	2.161045	2.645313	11.173636
H	1.672637	1.519628	10.200144
H	0.948664	2.909057	10.215841
H	2.713106	−0.515618	3.480895
H	2.988495	−1.986933	3.970553
H	1.574674	−0.826947	5.557745

H	2.897128	−1.311423	6.239955
H	4.628209	1.252822	4.047043
H	5.910950	0.745814	4.783241
H	1.419526	−1.083534	8.822583
H	−2.844874	−2.356024	9.777784
H	−2.915736	−3.062816	8.379595
H	−4.216149	−2.472709	9.029145
H	5.100376	−1.363610	4.859815
H	5.074022	−0.856226	3.376944

Listing 4: $[C_{17}H_{28}NO_2]^+$ cartesian coordinates (xyz) optimized at DFT/M062-X/def2-TZVP

O	0.644265	9.229910	3.983520
H	0.010670	9.236820	3.259030
O	4.005850	14.590500	5.484470
N	3.169680	8.898480	7.146780
H	3.150940	9.925070	7.085600
C	1.913560	8.798310	3.499310
C	2.175780	11.412600	5.401340
H	1.184930	11.021100	5.599480
C	2.314370	9.583460	2.246430
H	2.270790	10.650700	2.467930
H	3.356430	9.343090	2.008520
C	0.989171	6.973160	1.986400
H	−0.053531	7.214090	2.220430
H	1.013810	5.899820	1.795750
C	1.873230	7.293960	3.191060

H	2.892670	6.949020	2.987800
H	1.504870	6.751430	4.064450
C	4.665010	12.468400	4.801940
H	5.627640	12.901500	4.565490
C	3.668890	13.301900	5.317380
C	4.597060	8.480560	7.128000
H	4.633680	7.393350	7.100270
H	5.084370	8.850010	8.026630
H	5.075940	8.902540	6.249300
C	3.159890	10.571000	4.886110
C	2.496180	8.480040	8.404130
H	1.480600	8.866980	8.404020
H	3.049770	8.874080	9.252660
H	2.480910	7.392790	8.440850
C	2.911420	9.095430	4.650430
H	3.857680	8.640020	4.347040
C	1.438130	9.249090	1.040180
H	1.793120	9.808490	0.174225
H	0.411103	9.593560	1.210700
C	2.418350	8.419960	5.935720
H	1.369150	8.662070	6.099780
H	2.533760	7.337140	5.913370
C	3.020690	15.488600	5.962720
H	2.165480	15.526700	5.284420
H	3.496940	16.463200	6.002060
H	2.686080	15.205600	6.963450
C	1.431590	7.751930	0.752510

H	2.440140	7.435150	0.467533
H	0.778965	7.528600	−0.091875
C	4.408650	11.128600	4.595560
H	5.189590	10.502100	4.176770
C	2.418510	12.764600	5.620370
H	1.626050	13.387900	6.008020
